# Attention and the Speed of Information Processing: Posterior Entry for Unattended Stimuli Instead of Prior Entry for Attended Stimuli

**DOI:** 10.1371/journal.pone.0054257

**Published:** 2013-01-30

**Authors:** Katharina Weiß, Frederic Hilkenmeier, Ingrid Scharlau

**Affiliations:** 1 Department of Psychology, Leuphana University Lüneburg, Lüneburg, Germany; 2 Department of Cultural Sciences, Paderborn University, Paderborn, Germany; 3 Inkubator, Leuphana University Lüneburg, Lüneburg, Germany; University of Groningen, The Netherlands

## Abstract

Why are nearly simultaneous stimuli frequently perceived in reversed order? The origin of errors in temporal judgments is a question older than experimental psychology itself. One of the earliest suspects is attention. According to the concept of prior entry, attention accelerates attended stimuli; thus they have “prior entry” to perceptive processing stages, including consciousness. Although latency advantages for attended stimuli have been revealed in psychophysical studies many times, these measures (e.g. temporal order judgments, simultaneity judgments) cannot test the prior-entry hypothesis completely. Since they assess latency differences between an attended and an unattended stimulus, they cannot distinguish between faster processing of attended stimuli and slower processing of unattended stimuli. Therefore, we present a novel paradigm providing separate estimates for processing advantages respectively disadvantages of attended and unattended stimuli. We found that deceleration of unattended stimuli contributes more strongly to the prior-entry illusion than acceleration of attended stimuli. Thus, in the temporal domain, attention fulfills its selective function primarily by deceleration of unattended stimuli. That means it is actually posterior entry, not prior entry which accounts for the largest part of the effect.

## Introduction

Processing of temporal information is crucial to human life. It is involved in a wide range of experiences and behaviors (cf. [1]), for instance in biological rhythms, speech, and control of motor behavior. Veridical processing of temporal information seems vital and adaptive because deficits in temporal information-processing accompany many neurological, psychological, and developmental disorders occasionally causing severe difficulties in interactions with the environment (e.g., neglect: [2]; visual extinction: [3]; aphasia: [4]; attention deficit hyperactivity disorder: [5]; schizophrenia: [6] and dyslexia: [7]). Nevertheless, errors in temporal judgments are surprisingly common and not restricted to patients with neurological, psychological or developmental disorders but occur frequently in temporal judgments of the normal population. For instance, two physically simultaneous stimuli are often perceived as successive [8]–[11] and the order of two rapidly succeeding stimuli is frequently reversed (e.g., [9], [12]–[16]). The source of these and other temporal errors aroused interest even before the beginning of experimental psychology itself [9], [17]–[19] and was for the first time systematically investigated in the field of astronomy.

### 1.1 Temporal Errors in Astronomy

In 1796 Nevil Maskelyne, Astronomer Royal at Greenwich observatory, dismissed his assistant David Kinnebrook because he deviated from Maskelyne himself by 800 ms in estimating the moment in time when a star crossed a wire on the Greenwich telescope, a stellar transit. Since the method of observation, the “eye and ear method”, was assumed to be eight times as accurate, this deviation was severe. However, its theoretical importance remained unnoticed until the 1820s when Bessel systematically investigated judgments of stellar transits made by several well-trained astronomers and found even larger deviations. Bessel and other astronomers formalized such interindividual deviations in so called *personal equations* (e.g., [17], [19]).

But what is the origin of these large deviations between astronomic observers? In the eye and ear method, an observer begins to count the second beats on a clock when a star approaches one of the vertical wires of a telescope. He remembers the spatial positions of the star at the beat just before and just after the star crosses the wire. Then, the remembered spatial distances from these positions to the wire are translated into a temporal estimate of the moment in time at which the star crossed the wire (for a more detailed description of the eye and ear method see [19] or [18]). Although there are doubtlessly several sources causing personal equations – such as differences in neural transmission times for audition and vision (e.g., [8], [20], impaired recollection of the stars’ spatial positions or rounding errors in estimating a star’s transit [18] –, the most frequently blamed source is attention, e.g., [8], [9], [19], [21], [22]. Assuming that allocating attention to a stimulus is a precondition for its conscious perception and takes time (e.g., [23]), personal equations can be accounted for by differences in the allocation of attention towards the visual and auditory modality. Supposing Maskelyne primarily paid attention to vision – the position of the star –, whereas Kinnebrook primarily paid attention to audition – the clock’s beats –, they had to perceive a stellar transit at different points in time. This gives the stimulus in the primarily attended modality (star or clock beat) a headstart into following stages of information processing.

### 1.2 Attention as Source of Errors in Temporal Judgments

Thus attention is an old suspect regarding errors in temporal judgments. The facilitating influence of attention on temporal information processing has become known as the *notion of prior entry* (e.g., [13]–[15], [19], [24]–[27]). According to this explanation, attention leads to *acceleration* of attended stimuli and consequently to their “prior entry” to perceptive processing stages, including consciousness. This acceleration of attended stimuli would be accompanied by reversals of temporal order and errors in judgments of simultaneity. Prior-entry effects have been revealed many times within and between modalities (e.g. vision: [13], [24], [26]–[28]; audition: [29]; tactile modality: [10], [30]; bimodal: [22]; for an overview see [31]). Yet, the most frequent methods for assessing prior-entry effects – temporal order judgments (TOJ) and simultaneity judgments (SJ) – do not allow a complete test of the prior-entry hypothesis.

In both tasks, two target stimuli – for instance a click and a flash – are presented in fast succession or simultaneously. Two factors are varied between experimental trials, the temporal interval between the stimuli (stimulus onset asynchrony, SOA) and whether attention is directed to one of the stimuli or not. The observers’ task is either to judge which of the two stimuli appeared first (TOJ) or whether both stimuli appeared simultaneously (SJ). The prior-entry effect is operationalized as shift in the *point of subjective simultaneity* (PSS). The PSS usually corresponds to objective simultaneity if attention is not manipulated and is shifted to a temporal interval at which the unattended stimulus objectively leads the attended one if attention is directed. The PSS is either represented by the temporal interval at which both stimuli are most frequently judged as simultaneous (SJ, e.g., [10]) or the temporal interval at which both order judgments are given equally often (TOJ, e.g., [13]). In these tasks, prior-entry effects thus represent *relative* processing advantages for attended in comparison to unattended stimuli. However, as stated above, the prior-entry hypothesis goes beyond the prediction of a relative processing advantage. As its name indicates, it implies that processing of attended stimuli is accelerated. Although relative processing advantages for attended stimuli are traditionally interpreted as acceleration of attended stimuli (prior-entry hypothesis), they could just as easily be explained by deceleration of unattended stimuli (posterior-entry hypothesis). Deceleration of unattended stimuli seems to be even more plausible because veridical perception of attended – thus possibly (action-) relevant information – should be most beneficial: Imagine a botanist trying to catch a rare butterfly in a cloud of common butterflies. It would be most helpful to perceive the rare butterfly veridically in time, whereas processing of the common butterflies would be slowed down. This possible interpretation seems to have escaped most researchers. Only two studies found indirect support for a posterior-entry hypothesis: Spence, Nicholls and Driver [32] found that directing attention to a specific modality primarily led to deceleration of discrimination latencies in unattended modalities. In their visual prior entry study Shore et al. [28] found evidence for prior entry as well as posterior entry in rare simple RT trials which were intermixed with temporal order judgment trials. RTs were faster for valid cues than for invalid cues in comparison to a neutral baseline.

The aim of the present study is a genuine test of the prior- vs. posterior-entry hypothesis. Therefore we developed a new paradigm providing *separate* estimates of processing (dis)advantages for attended and unattended stimuli in comparison to a baseline condition without the manipulation of attention. It relies on one of the oldest prior-entry paradigms – the complication clock of Wilhelm Wundt [9]. In a complication experiment, observers watch the movement of a clock’s hand while waiting for a single event in another sense modality, for example a sound. The observer judges the hand’s position at the moment the sound appears. Usually, hand positions that lie objectively before the appearance of the sound are reported (e.g., [9], [25]; for more recent studies using versions of complication paradigms see [33]–[36]). Prior entry provides a probable explanation for this finding as paying attention to the sound would lead to its earlier perception. Recently, Carlson, Hogedoorn and Verstraten [37] adapted the complication clock paradigm to assess the speed of visual attention shifts. Observers watched an array of clocks with rotating hands and reported the time of one of these clocks indicated by a peripheral or central cue at the moment when this cue appeared**.** Clock times revealed latencies of 140 ms for an attention shift initiated by a peripheral cue and 240 ms for an attention shift initiated by a central cue. More recently, Hogendoorn, Carlson and Verstraten [38] used the same paradigm to assess the latency of attentional selection. Additionally, Carlson and colleagues used adaptations of this task to assess attention shifts and attentional dwell time in attentive tracking [39] or the costs of dividing attention [40]. Furthermore Keetls and Vroomen [41] used a very similar technique to assess temporal ventriloquisim. Observers saw an array of clocks with revolving hands and reported the time of a specific clock when it was cued. Presenting an irrelevant tone 100 ms before the spatial cue shortened the difference between actual and reported time. Thus, the tone shifted the temporal position of the cue (temporal ventriloquism).

**Figure 1:**
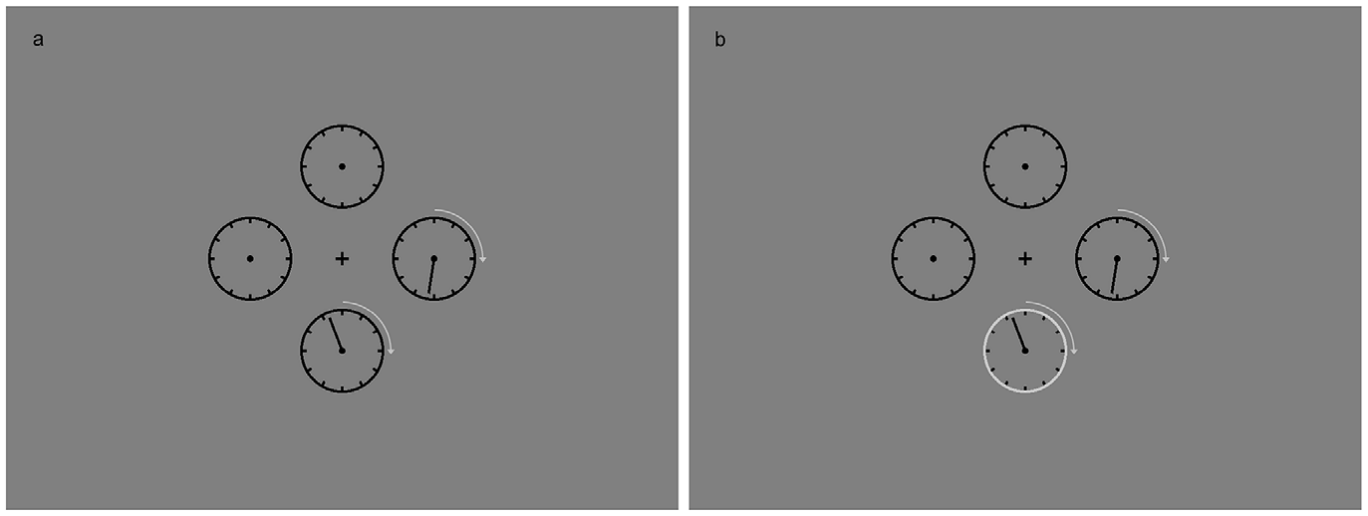
Experimental Setup. In two of four empty clocks moving hands appeared with an SOA of either 0 ms, 34 ms, 68 ms, 136 ms or 272 ms. In two thirds of the trials, either the first or second hand’s clock was cued by flashing the clock’s rim (here indicated by a brighter rim of the clock, Panel b). In the remaining third of trials (baseline condition) none of the clocks was cued (Panel a).

To assess posterior and prior entry we combined the TOJ task with a clock paradigm adapted from Carlson et al. [37]. Observers watched an array of empty clocks (Figure 1). After variable time, continuously moving hands appeared in two of these clocks. Two factors were varied over experimental trials: (1) the temporal interval between hands’ onsets and (2) whether attention was directed to one of the clocks by a peripheral cue or not. The observers’ task was to judge which hand appeared first and to report the initially displayed time of both clocks**.** To this end they adjusted the clocks’ hands to the perceived position of their appearance. Compared to an ordinary TOJ task, the new paradigm has the advantage that time judgments provide separate estimates of advantages or disadvantages of an attended and unattended stimulus.

Supposing that prior-entry effects are due to acceleration of attended stimuli, the attended clock’s time will be perceived as earlier than the same clock’s time if none of the clocks is attended to. Perceived time of the unattended clock will be unaffected. By contrast, if prior-entry effects are exclusively due to deceleration of unattended stimuli, perceived time of the attended clock will be unaffected, whereas the unattended clock’s time will be perceived as later than the same clock’s time if attention is not manipulated. Finally, if both mechanisms cause prior-entry effects, the attended clock’s time will be perceived as earlier and the unattended clock’s time as later than if attention is not manipulated.

According to Carlson and coworkers [37]–[40] as well as Vroomen and Keetels [41] the clock task provides a rather direct measure of perceptual latency or latencies of an attention shift; the clock times should thus be closely related to perceptual latency measured by the temporal order judgment in prior-entry research. (Note that although most researchers seem to agree that some higher cognitive processing such as attention or consciousness is a precondition for temporal order judgments, e.g. [16], [23], [42] some of these judgments can be made on the basis of onset detection only [43]). However, even if the strong and debatable presupposition that the clock task provides a rather direct measure of perceptual latency or latencies of an attention shift is not shared and the clock task is interpreted as a spatial task, it will still answer our question. The clock task may be regarded as a variant of a spatial task used for studying the Fröhlich effect (e.g. [44]–[47], for a recent overview see [48]). In this misperception, the perceived initial position of a quickly moving stimulus abruptly entering the visual field is mislocalized in the direction of its motion. Because of two reasons this basically spatial misperception is likewise appropriate to answer the question of the present paper. Firstly, it can reveal a pattern of facilitation and inhibition for the cued and uncued clock which is what we are interested in. Secondly, the spatial measures are closely related to temporal values in prior entry on the basis of the attentional account [23], [24] of the Fröhlich effect: In order to report the spatial position of a (moving) stimulus, an observer has to complete an attention shift towards the position of the stimulus. The initial appearance of a moving stimulus will trigger an attention shift towards its location. Since the stimulus is moving while attention is under way, the observer will report a later position of the moving stimulus than its actual position. In accordance with this attentional account, several studies [47], [49], [50] found that the size of the Fröhlich effect was reduced if the location of the moving stimulus was cued in advance. In connection with the aim of the present study – measuring prior entry respectively posterior entry effects with a clock paradigm – it is important to note that the same attentional model [23], [47] has been used to explain prior-entry effects.

Translated into the spatial terms of the clock task, if prior-entry effects are due to acceleration or facilitation of the attended stimulus, the spatial mislocalization of the attended clock should be reduced by spatial attention – as indeed reported by [47], [49], [50]. By contrast, if prior-entry effects are exclusively due to deceleration or inhibition of the unattended stimulus, the misperception of the clock from which attention is drawn away should increase in comparison to the baseline. If, finally, both mechanisms contribute to the effect, Fröhlich effects on the attended clock will decrease and those on the unattended clock will increase.

Let us underline again that our argument does not depend on the assumption that reported clock times actually provide a measure for perceptual latencies or that the latency of an attention shift can be computed from these clock times although such claims have indeed been made by Carlson and colleagues [37]–[40] as well as Vroomen and Keetls [41]. Since we compare conditions in which attention is manipulated (attended clock, unattended clock) with conditions in which attention is not manipulated, all differences between these conditions should reflect that part of the Fröhlich effect which is prone to attention.

Additionally, possible acceleration and deceleration effects should be modulated by the degree of spatio-temporal interference caused by direction of attention or by the temporal interval between the targets. Because the temporal interval between attention directing cue and cued target in TOJs is fixed, the cue can direct attention relatively unimpaired if the first target (T1) is cued but not if the second target (T2) is cued. In the latter case, T1 appearance interferes with attentional allocation by the cue to T2. Consequently spatio-temporal interference should weaken possible acceleration of the attended T2 because the cue and T1 compete for attentional capture. The influence of spatio-temporal interference on possible deceleration of the unattended T1 is less clear. On the one hand, deceleration might be weakened too, whereas on the other hand, interference might enhance deceleration of the unattended T1, because deceleration of irrelevant information would be most convenient under high temporal competition. Empirical findings of attentional effects that are restricted to high external noise conditions support this latter assumption. Here, attentional facilitation of attended locations is primarily achieved by noise reduction instead of signal enhancement (e.g., [51], [52]).

The temporal interval between T1 and T2 appearance can influence observed effects as well. Spatio-temporal interference between the targets is larger for small temporal intervals because two targets in close temporal proximity compete more likely for attentional capture. For instance, Gibson and Egeth [53] showed for inhibition of return – inhibition of a cued location after long cueing intervals above 300 ms – that this attention-related phenomenon depends on spatio-temporal interference caused by the cue, as well as the size of temporal interval between the targets. Inhibition of return was present if T2 was cued but suppressed for small temporal intervals if T1 was cued. It could only be demonstrated if T1 was presented more than 100 ms before T2.

## Methods

### 2.1 Participants

Twenty students (twelve female, eight male) of Paderborn University took part in the experiment. All had normal or corrected to normal vision verified by a simple test. Participants received either a financial reward of 6 Euro or participated for course credit.

#### 2.1.2 Ethical statement

Before conducting the experiment, participants read and signed an informed consent. All data was de-identified and analyzed anonymously. Since the Founding Agency “Deutsche Forschungsgemeinschaft” did not request an ethical approval and Paderborn University has no board to review ethical standards, no ethical approval for the experiment was obtained. This proceeding is in line with the ethical guidelines of the “Deutsche Gesellschaft für Psychologie*”* which states on ethical approval :“ C.III.2 Formal ethical approval. If research projects need a formal ethical approval, psychologists provide precise information about their research project. They only begin with the research project after receiving the approval. They conduct their research project in line with the approved proceeding.” Which kind of research projects need an ethical approval is clarified by the ethical board of the *Deutsche Gesellschaft für Psychologie:* “In general a request for ethical approval of a psychological project is addressed to ethical board if a research funding body (i.e. DFG, VW-Foundation, FP7 of the EU, federal ministries or federal state ministries, foundations, universities) requests an ethical approval for the project. Such a request is usually to be expected if human participants are put at risks or for studies in which human participants are not fully aware about the aims and procedures of the study.” These conditions do not apply to the present experiment. Additionally, the experiment was conducted respecting the ethical standards for research with human participants of the American Psychological Association.

### 2.2 Apparatus

Participants sat in a dimly lit room with viewing distance fixated at 57 cm by a chin rest. The center of the monitor was at eye level. The experiment was presented on a screen of a 19 inch cathode ray tube monitor, stimuli were dark gray (26.6 cd/m2) on a light gray background (93.4 cd/m2). The monitor’s refresh rate was 60 Hz and its resolution was set to 1024 × 768 pixels. The experimental program was written in Matlab R2009a and made use of the Psychtoolbox-3 [54], [55].

### 2.3 Stimuli

Four clocks lacking their hands were placed a distance of approximately 5.7° around fixation. Diameter of the clocks was 2.5°. Target stimuli were two lines (2.1° length) serving as moving clock hands. They appeared in two of the four clocks in each trial. A bright flash of one of the clocks’ rims, which was turned off after 34 ms, served as a cue.

### 2.4 Procedure

At the beginning of each trial, a fixation cross was presented in the middle of the screen with four symbolic clocks lacking their hands symmetrically grouped around it. After a random interval, the first hand (T1) appeared in one of the clocks at a randomly chosen position, moving continuously in clockwise direction with a speed of three degrees in 17 ms. After a variable SOA (0 ms, 34 ms, 68 ms, 136 ms or 272 ms), a second hand (T2), moving with the same speed and direction, appeared. Both hands were turned off simultaneously after a maximal turn of three quarters of the clock. In one third of trials, the T1 clock was cued by a bright flash of its rim (T1 cue). The SOA between cue and target (cueing SOA) was 166 ms. In a second third of trials, the T2 clock was cued (T2 cue). In the remaining third, none of the clocks was cued (no cue). After each trial, observers made a TOJ by choosing the clock in which the first hand appeared using the arrow keys. They then adjusted both hands until they displayed their perceived onset position, using the arrow keys. 240 trials with the factors Cue 3×(no cue, T1 cue, T2 cue) 5×Target SOA (0 ms, 34 ms, 68 ms, 136 ms, 272 ms) were presented randomly. Each combination of experimental conditions was repeated 16 times. A session had a mean duration of 45–50 minutes.

## Results

### 3.1 Temporal Order Judgments

**Figure 2:**
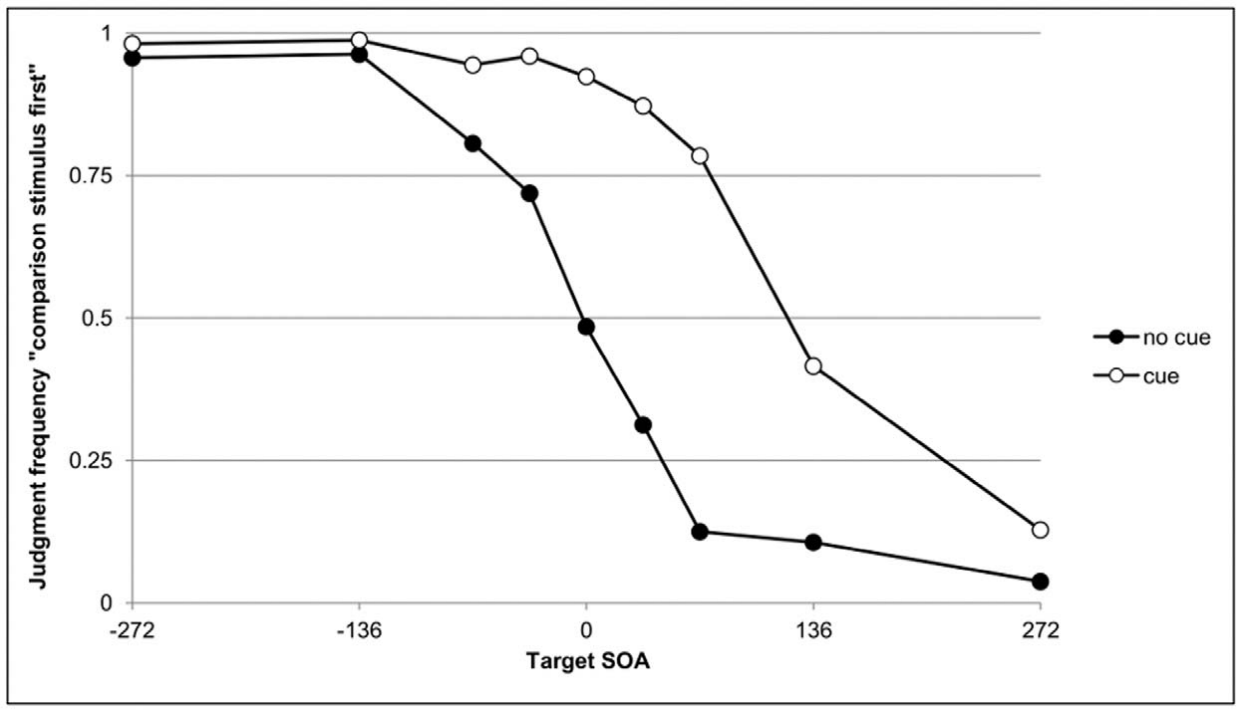
Order judgments. Figure 2 shows the judgment frequencies for the order judgment “comparison stimulus first” for all target SOAs (−272 ms, −136 ms, −68 ms; −34 ms; 0 ms; 68 ms; 136 ms; 272 ms) separately for the uncued baseline condition (black circles ) and the cued condition (white circles). In cued trials, the cued stimulus is defined as comparison stimulus whereas the uncued stimulus defines the standard stimulus. In uncued trials the labels comparison and standard stimulus are randomly assigned. The horizontal shift of the judgment frequencies demonstrates a prior-entry effect of 119 ms.

TOJs were analyzed first because an analysis of clock times would be obsolete without a replication of the standard prior-entry effect in the present experimental paradigm. For constructing psychometric functions, target SOAs except SOA zero were divided into SOAs in which T1 was cued (negative SOAs) and SOAs in which T2 was cued (positive SOAs). For uncued trials, positive and negative SOAs were assigned randomly. For each of the resulting nine target SOAs, order-judgment frequencies were counted for cued and uncued trials separately. Figure 2 displays the order-judgment frequencies, which were approximated by logit analysis [56]. Two parameters were derived from each psychometric function, the PSS and the difference limen (DL), as measure of discrimination accuracy. DL values were assessed because prior-entry effects are sometimes accompanied by changes in temporal discrimination accuracy. Whereas Stelmach and Herdman [24] found better discrimination accuracy under attentional allocation, we found consistently that discrimination accuracy becomes worse under these circumstances [26], [27]. DL is taken as half of the innerquartile range of the psychometric function. Smaller DL values thus indicate better discrimination accuracy.

Three participants’ data were excluded from further statistical analysis because they showed flat psychometric functions in at least one experimental condition. PSS differences between uncued and cued trials revealed a substantial prior-entry effect of 119 ms, *t*(16) = 10. 67, *p*<.001, *d* = 2.66. A comparison of DL values revealed no difference between cued (*M* = −48 ms) and uncued (*M* = −44 ms) conditions, *t* <1.

### 3.2 Clock Times

**Figure 3:**
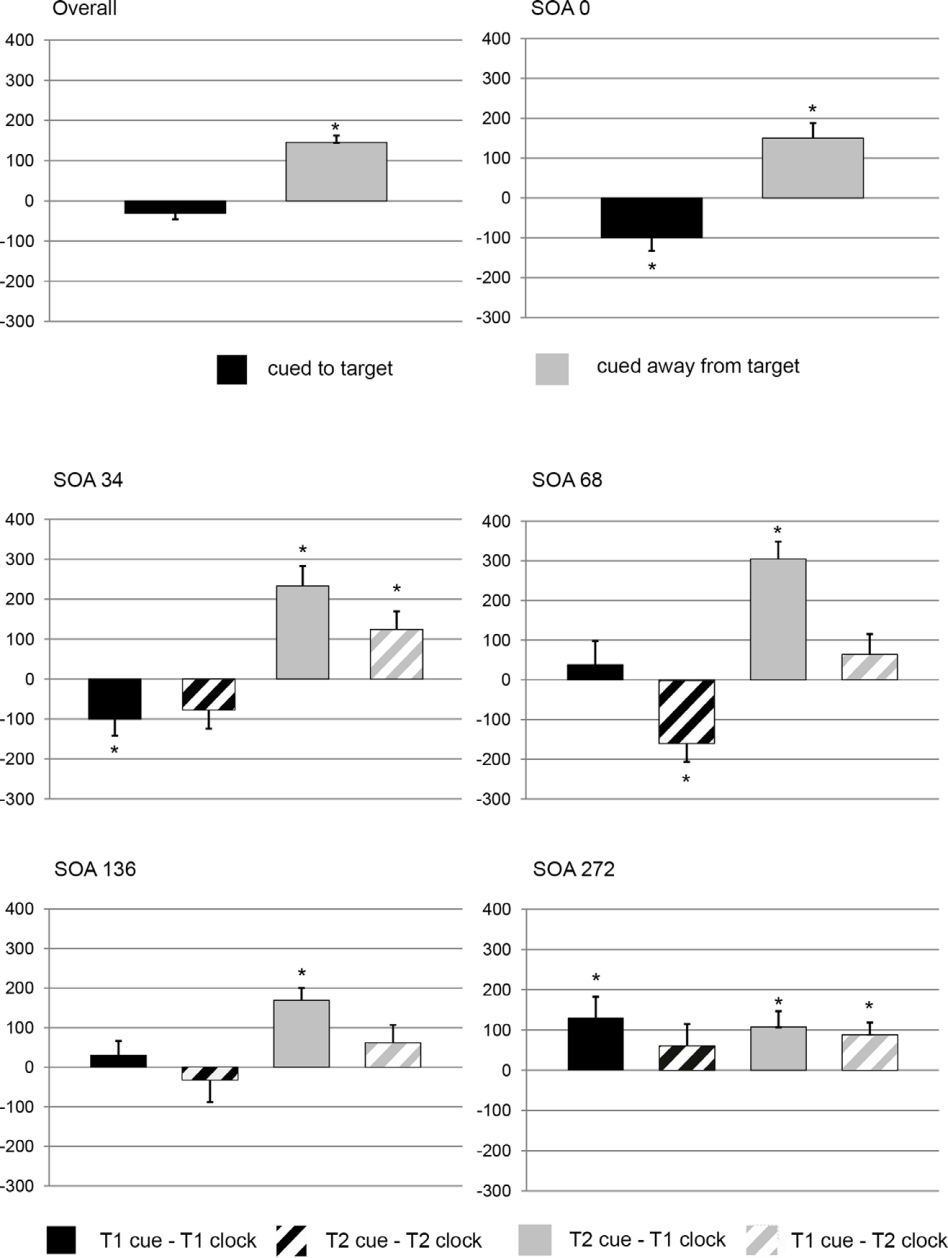
Clock times. Figure 3 shows the results of the time judgments. Effects significantly different from zero were marked with an asterisk, all ps at least <.05. Error bars indicate the standard error of the mean (SEM).

Reported spatial positions of clock hands were translated into time values. To standardize time judgments, difference-values between subjective and objective times were calculated for each combination of clock (T1 clock, T2 clock), cueing condition (no cue, T1 cue_,_ T2 cue) and target SOA (0 ms, 34 ms, 68 ms, 136 ms, 272 ms). In accordance with the attentional mechanisms and the experimental rationale discussed in the Introduction, we took values from no-cue conditions as baseline to remove the targets’ perceptual latencies if attention is not manipulated, as well as any other deviation from objective clock times which is not due to attention, e.g. strategic biases. Therefore, no-cue conditions difference-values were subtracted from the respective cued-conditions difference-values. Negative values denote acceleration effects, whereas positive values denote deceleration effects in comparison to the respective uncued baseline-condition (Figure 3).

To test for possible acceleration or deceleration effects we computed repeated measures ANOVAs, as well as t-tests against zero; only significant effects will be reported. Since partial eta-square (*η_p_^2^)* measured in within-subjects designs is not comparable with *η_p_^2^* assessed in between-subjects designs, we used *eta-square general (η_G_^2^;* [57], [58]) as measure of effect size to ensure good comparability between studies. An ANOVA with factors Attention (cued to target/cued away from target)×Target (T1 clock, T2 clock)×SOA (0 ms, 34 ms, 68 ms, 136 ms, 272 ms) was conducted. Since targets appear simultaneously at SOA zero, T1 and T2 were defined arbitrarily. Including SOA zero provides the more conservative test for factor Target because at this SOA T1 and T2 clock times should not differ. A main effect of Attention, *F*(1,16) = 66.903, *p*<.001, *η_G_^2^* = .19, was due to a large – both in numerical and in effect size – deceleration effect (*M* = 145 ms) for the uncued clock, *t*(169) = 9.75, *p*<.001, *d* = 0.75 and a smaller acceleration effect (*M* = −31 ms) for the cued clock, *t*(169) = −1.92, *p* = .056, *d* = 0.14. A main effect of Target, *F*(1,16) = 10.39, *p*<.01, *η_G_^2^* = .05, demonstrates that time on T1 was postdated in comparison to time on T2 (*M* = 98 ms vs. *M* = 17 ms). Interactions were found for Attention×SOA, *F*(3,52) = 9.23, *p*<.001, *η_G_^2^* = .07 and Attention×Target, *F*(1,16) = 8.76, *p*<.01, *η_G_^2^* = .004. The interaction SOA×Target was marginally significant, *F*(3,53) = 2.45, *p* = .07, *η_G_^2^* = .04. Since different degrees of spatio-temporal interference probably modulate possible acceleration and deceleration effects, we analyzed time judgments separately for each target SOA (target interference) and cue-target combination (cue-target interference).

*SOA 0*: An ANOVA with the single factor Attention revealed a main effect of Attention, *F*(1,16) = 29.52, *p*<. 001, *η_G_^2^* = .63 which was due to a large deceleration effect (*M* = 150 ms, *t*(16) = 3.97, *p*<.001, *d* = 0.99) for the uncued clock and a smaller acceleration effect (*M* = −99 ms, *t*(16) = −2.98, *p*<.01, *d* = 0.75) for the cued clock.

*SOA 34*: An ANOVA with the factors Attention×Target revealed a main effect of Attention, *F*(1,16) = 48.77, *p*<.001, *η_G_^2^* = .40, which was due to an acceleration effect (*M* = −89 ms) for the cued clock, *t*(33) = −2.87, *p*<.01, *d* = 0.50 as well as an even larger deceleration effect (*M* = 179 ms) for the uncued clock, *t*(33) = 5.1, *p*<.01, *d* = 0.89. The interaction Attention×Target was marginally significant *F*(1,16) = 4.10, *p* = .06, *η_G_^2^* = .04. T-tests against zero revealed an acceleration effect for the T1 cue on T1 (*M* = −100 ms) and significant deceleration effects for the T1 cue on T2 (*M = *124 ms) and the T2 cue on T1 (*M = *232 ms), all *ps* <. 05.

*SOA 68*: An ANOVA with the factors Attention×Target revealed a main effect of Attention *F*(1,16) = 31.51, *p*<.001, *η_G_^2^ = *.39, which was primarily due to a large deceleration effect for the uncued clock (*M* = 184 ms, *t*(33) = 4.72, *p*<.001). A main effect of Target demonstrates that time on T1 was postdated in comparison to time on T2 (*M* = 171 ms vs. *M* = −47 ms). T-tests against zero revealed an acceleration effect for the T2 cue on T2 (*M* = −160 ms) and a large deceleration effect (*M* = 305 ms) of the T2 cue on T1, both *ps* <.01.

*SOA 136*: An ANOVA with the factors Attention×Target revealed a main effect of Attention, *F*(1,16) = 11.11, *p*<.01, *η_G_^2^* = .14, which is due to a deceleration effect for the uncued clock (*M* = 116 ms), *t*(33) = 4.06, *p*<.001, *d* = 0.71. The marginally significant main effect of target, *F*(1,16) = 3.08, *p = *.10, *η_G_^2^* = .08 demonstrates that time on T1 was postdated in comparison to time on T2 (*M = *100 ms vs. *M* = 14 ms). T-test against zero revealed a deceleration effect of (*M = *170 ms) for the T2 cue on T1, *t*(16) = 5.55, *p*<.001, *d* = 1.39.

*SOA 272*: An ANOVA with the factors Attention×Target revealed no significant effects, all *Fs* <1.1. T-tests against zero revealed significant deceleration effects for T1 cue on T1 (*M* = 130 ms), T2 cue on T1 (*M = *108 ms) and T1 cue on T2 (*M* = 88 ms), all *ps* <.05.

Thus the binary TOJ revealed a relative latency advantage of 119 ms for attended stimuli and no influence of attention on discrimination accuracy. Clock times revealed that the relative latency advantage for attended stimuli in the TOJ is primarily due to deceleration of unattended stimuli (145 ms) with a small contribution of acceleration of attended stimuli (31 ms). Furthermore, deceleration of unattended stimuli was consistently found for all target SOAs, whereas acceleration of attended stimuli was found only for the three smallest SOAs (0 ms, 34 ms, 68 ms). At the longest SOA (272 ms), deceleration effects were even obtained for attended stimuli. Additionally, deceleration effects were largest for high spatio-temporal interference between cue and uncued target (T2 cue on T1) whereas acceleration effects were more often revealed under conditions in which the cue could capture attention unimpairedly (T1 cue on T1).

## Discussion

Despite its eponymous, traditional interpretation, the temporal illusion which has been known for over 150 years as the *prior-entry* effect is primarily due to deceleration of an unattended stimulus (145 ms) instead of acceleration of an attended stimulus (31 ms). Although several researchers suggested that prior-entry effects could just as well be due to deceleration of unattended stimuli ([15], [24]; [22], p. 823; [59], p. 103), the possibility of a *posterior-entry* effect has mostly been ignored in the field of prior entry. It is therefore a crucial question how posterior entry could be reconciled with prior-entry models. While it provides a serious challenge for some prior-entry models (e. g. the asynchronous-updating model, [23])**,** there are other models which can be easily adjusted to reflect a posterior-entry mechanism: the *temporal-profile model* (TPM, [24]) and the temporal order models adapted by Schneider and Bavelier [60], the deterministic decision model and perceptual moment respectively triggered moment model. In the TPM, temporal order is detected by comparing whole temporal profiles of stimuli, not only their arrival times. The first peak in the resulting difference function denotes which stimulus is perceived first. In the original version of the TPM, attention accelerates processing of an attended stimulus by sharpening its temporal profile. But, as already admitted by Stelmach and Herdman, the TPM could also explain prior-entry effects by decelerated processing of an unattended stimulus via broadening the unattended stimulus’ profile. In their original formulation, all temporal order models described by Schneider and Bavelier could explain prior entry by an acceleration of transmission time of attended stimuli. Therefore an attended stimulus would arrive earlier at the respective decision mechanism and would more likely be perceived as the first stimulus. Such a mechanism could easily be adapted to explain posterior-entry under the assumption that attention decelerates the transmission time of the unattended stimulus: Therefore the unattended stimulus would arrive later at the respective decision mechanism and would less likely be perceived as the first stimulus. In contrast to the described temporal-order models, the AUM only provides a prior-entry explanation. Attention is needed to transfer information represented on feature maps into an internal model which is a necessary precondition for awareness. Directing attention to a spatial location before target appearance would accelerate the target’s transfer into the internal map because otherwise the target would have had to initiate the time consuming attention shift by itself.

Furthermore, posterior entry is in accordance with the idea that attention facilitates information processing not only by *signal enhancement* but also by *exclusion of external noise* (e.g., [51]). Interestingly, we found particularly pronounced deceleration effects on unattended stimuli for conditions in which spatio-temporal interference between cue and uncued target is high (T2 cue). This agrees with empirical findings implicating that external noise reduction underlies attentional facilitation especially under high external noise [51], [52].

It is an interesting question whether posterior entry is also accountable for cross-modal prior-entry effects (e.g., [22], [61]). The neural mechanisms underlying attention to a location and attention to a sensory modality might be different [62]. In consequence the mechanisms underlying intra-and crossmodal prior-entry effects would be different as well. Therefore it seems possible that intramodal prior-entry effects, as established in the present study, are primarily due to posterior entry whereas crossmodal prior-entry effects are actually due to prior entry. We have to leave this topic to future research. Summing up, in the temporal dimension, (spatial) attention fulfills its selective function primarily by deceleration of unattended, probably irrelevant information, thereby leading to their *posterior entry* into higher stages of information processing.
